# Developmental Behavioural Plasticity and DNA Methylation Patterns in Response to Predation Stress in Trinidadian Guppies

**DOI:** 10.1111/mec.17831

**Published:** 2025-06-13

**Authors:** Janay A. Fox, Simon M. Reader, Mélanie F. Guigueno, Rowan D. H. Barrett

**Affiliations:** ^1^ Department of Biology McGill University Montreal Quebec Canada

**Keywords:** behavioural plasticity, development, epigenetics, fish, *Poeciliid*

## Abstract

Early‐life experiences can predict the environments experienced later in life, giving individuals an opportunity to develop adaptive behaviour appropriate to a likely future environment. Epigenetic mechanisms such as DNA methylation (DNAm) have been implicated in developmental behavioural plasticity; however, studies investigating this possibility are limited in taxonomic breadth and ecological relevance. We investigated the impact of early‐life exposure to predation stress on behaviour and DNAm in the brains of Trinidadian guppies ( *Poecilia reticulata* ). We exposed guppies throughout development to either an alarm cue (conspecific skin extract), inducing predation stress, or a control cue (water) for 8 weeks and then raised them to adulthood under identical conditions. Then, we conducted two behavioural assays, an open‐field and a grouping test, before performing whole‐genome bisulfite sequencing on whole brains. Guppies exposed to the alarm cue during development exhibited increased grouping (shoaling) in adulthood compared to those exposed to the control treatment, but there were no detectable impacts on activity, boldness, or exploratory behaviour. We also identified stable shifts in brain DNAm in response to developmental alarm cue exposure in genes involved in behavioural regulation. Some differentially methylated sites were significantly associated with shoaling propensity in both males and females. Additionally, males and females differed in the magnitude of DNAm responses and the genes impacted, suggesting distinct roles for DNAm between the sexes. This study shows how early‐life predation stress can induce behavioural changes in adulthood and that shifts in neural DNAm could be an underlying mechanism responsible for these changes.

## 1 Introduction

Differences in early life experiences have been shown to have long‐lasting phenotypic impacts in a wide range of species (Beldade et al. 2011; Snell‐Rood 2013). For example, maternal diet affects coat colour variation in laboratory mice ( *Mus musculus* ; Waterland and Jirtle 2003), rearing exposure to hypoxia stimulates gill development in blue gourami fish ( *Trichopodus trichopterus* ; Blank and Burggren 2014), and increased early‐life food competition impacts foraging decisions later in life in the European starling ( *Sturnus vulgaris* ; Bloxham et al. 2014). This developmental plasticity has been conceptually distinguished from other types of plasticity, with a slower phenotypic response time and greater trait integration, and can involve shifts in morphology, life history, physiology and/or behaviour that may be permanent (Beldade et al. 2011; Snell‐Rood 2013). If early‐life environmental stressors reliably predict conditions that will be encountered later in life, then developmentally plastic responses could be used to adaptively shift adult phenotypes (Beldade et al. 2011; Snell‐Rood 2013). For example, rats exposed to chronic stress during adolescence exhibited long‐term changes in foraging behaviour that were beneficial to them when foraging under high‐threat conditions as adults (Chaby et al. 2015). Such adaptive behavioural plasticity could be especially beneficial where predation pressure varies. Alternatively, stressful early‐life experiences can challenge developmental processes and result in the expression of maladaptive plasticity. This can lead to a ‘silver spoon’ effect, found in many studies where animals born in less stressful conditions perform better later in life (reviewed in Lindström 1999; Mainwaring et al. 2023).

Predation pressure has strong impacts on behaviour (Lima and Dill 1990) particularly through the adoption of anti‐predator behaviours (Magurran 1990a; Riechert and Hedrick 1990; Stanford 2002). Since predation pressure varies spatially and temporally, behavioural plasticity is important for balancing anti‐predator behaviours with other fitness‐related activities such as reproduction or foraging (Lima and Dill 1990; Clinchy et al. 2013). Developmental behavioural plasticity can enhance fitness by allowing behavioural development to adjust to predation risks encountered in early life, assuming that this will represent risks encountered in later life. Accordingly, many species display developmental behavioural plasticity in response to predation (Ghalambor and Martin 2002; Dingemanse et al. 2009; Donelan and Trussell 2018). For example, exposure to predation pressure in adolescent laboratory rats decreased exploratory behaviour later in life (Adamec et al. 2001), freshwater snails ( *Lymnaea stagnalis* ) exposed to predatory fish kairomones during early life increased predator avoidance responses in adulthood (Dalesman et al. 2009), and damselfish ( *Pomacentrus wardi* ) exposed to predator cues as juveniles displayed risk‐averse behaviour as adults (Lönnstedt et al. 2012). Often the fitness consequences of such behavioural changes are not measured, but it is expected that these developmentally plastic behaviours would modify exposure to predation risk. For example, decreased exploration or increased risk‐averse behaviour could decrease exposure to predators. Despite the importance of developmental behavioural plasticity for allowing organisms to cope with environmental heterogeneity, studies investigating the underlying molecular mechanisms remain limited.

Recently, epigenetic mechanisms have emerged as a potential regulator of phenotype plasticity through changes in gene expression that are not associated with changes in the gene sequence (Feil and Fraga 2012). DNA methylation (DNAm), the addition of a methyl group on a cytosine typically in cytosine‐guanine dinucleotides (CpGs), is the most well‐studied epigenetic mechanism and plays a major role in gene expression (Jones 2012). Due to the environmentally responsive nature of DNAm, it is a prime candidate mechanism for developmental behavioural plasticity and has already been associated with environmentally induced behavioural variation (Weaver et al. 2004; Azzi et al. 2014; Saunderson et al. 2016). Indeed, recent studies have provided evidence indicating lasting, stable changes in DNAm can arise due to early life experiences and may play a role in the early life modification of adult behaviours (Weaver et al. 2004; Labonté et al. 2012; Zocher et al. 2020). In laboratory rats, differences in maternal care—which also result in differences in offspring stress responses (Francis et al. 1999; Weaver et al. 2004), neuroplasticity (Liu et al. 2000; Champagne et al. 2008), and learning (Liu et al. 2000; Lévy et al. 2003) that persist into adulthood (Champagne et al. 2003)—have been associated with changes in DNA methylation at the glucocorticoid receptor gene in the hippocampus (Weaver et al. 2004) as well as other broad methylome changes in the brain (McGowan et al. 2011). DNAm has also been implicated in developmental behavioural plasticity in fish. Mangrove killifish (*Kryptolebias marmoratus*) that were exposed to differing levels of environmental structure during development exhibited shifts in behaviour (such as activity and neophobia) and DNAm in the brain (Berbel‐Filho et al. 2020). In three‐spine stickleback ( *Gasterosteus aculeatus* ), differences in paternal care induced shifts in the expression of Dnmt3a, a DNA methyltransferase responsible for de novo methylation (McGhee and Bell 2014). Research on mice indicates that DNAm could be involved in responses to predation threat. For instance, mice given an acute exposure to cat predator cues show individual variation in behavioural coping styles that are associated with differences in DNAm (Bowen et al. 2014). Furthermore, neonatal mice exposed to a combination of different predator cues (adult male mouse, ferret, and cat) exhibit a sex‐specific increase in methylation at the 5‐hydroxytryptamine receptor 2A promoter in adulthood (Kigar et al. 2017). However, these mice studies are limited in scope as the first does not directly investigate developmental plasticity and the second only considers methylation at a single gene. Most studies exploring the role of DNAm in developmental behavioural plasticity have been done in mammalian systems and focus on environmental cues that are not experienced by natural populations or behaviours with unclear fitness ties. This limits our knowledge of the relevance of DNAm changes in nature. Therefore, there is a need for DNAm research that incorporates study systems with well‐characterised ecologically relevant environmental cues and behavioural shifts that have known fitness impacts. Additionally, many studies measure shifts in DNAm immediately after the developmental cue exposure, making it problematic to distinguish between developmental effects and recent experience.

The Trinidadian guppy ( *Poecilia reticulata* ) (hereafter ‘guppy’) provides a useful system for studying developmental behavioural plasticity due to the wealth of background knowledge on their evolutionary and behavioural ecology (Magurran 2005) and the wide range of behavioural plasticity they exhibit (Fox et al. 2024). In nature, guppies are exposed to a spectrum of predation pressures, with populations often divided by waterfalls that provide physical barriers separating low and high predation populations, leading to evolved differences in life history (Reznick and Endler 1982; Rodd and Reznick 1997), morphology (Johansson et al. 2004; Burns et al. 2009), colour patterns (Endler 1980) and behaviour (Seghers 1974; Brown et al. 2009; Elvidge et al. 2016). Additionally, guppies respond strongly to an ‘alarm cue’ that is released from conspecific skin damaged during a predation event (Brown and Godin 1999; Brown et al. 2010); this cue provides information regarding predation risk in their environment (Brown 2003) and can be used to induce predation stress (Elvidge et al. 2014; Stephenson 2016; Fan et al. 2022). Early life exposure to predation stress can lead to developmental shifts in behaviour in guppies that likely have impacts on fitness. For example, rearing with predation cues induced increased shoaling behaviour (Li et al. 2022), risk sensitivity (Krause and Liesenjohann 2012) and cognitive flexibility (Vila Pouca et al. 2021). The molecular mechanisms of this developmental behavioural plasticity have not been investigated in guppies; however, high and low predation guppies are known to differ in brain gene expression (Ghalambor et al. 2015; Reddon et al. 2022) suggesting epigenetic mechanisms could be playing a role.

In this study, we use guppies to investigate DNA methylation as a potential underlying mechanism of developmental behavioural plasticity. We hypothesised that exposure to alarm cue during early life would induce shifts in behaviour and DNAm in the brain. The behaviours we focussed on were shoaling, exploration (behaviour directed towards acquiring information in a novel environment (Burns et al. 2016)), boldness (propensity to take risks (Harris et al. 2010)), and activity, as these behaviours modify exposure to predation risk; therefore, shifts in these behaviours likely have fitness impacts. Accordingly, these behaviours have previously been shown to shift in guppies under high predation (Magurran and Seghers 1991; Harris et al. 2010; Krause and Liesenjohann 2012). We predicted that guppies exposed to alarm cue when juvenile would show increased shoaling and boldness, and decreased exploration and activity when adult, matching behavioural propensities seen in high predation guppies (Magurran and Seghers 1991; Harris et al. 2010; Burns et al. 2016). We also predicted that alarm cue‐exposed guppies would show shifts in neural DNAm in genes related to behavioural regulation and that differences in methylation would be associated with behaviour. The results of this work provide insights into the role of DNAm as a molecular mechanism underpinning developmental behavioural plasticity in fish and in ecologically relevant behaviours with consequences for fitness.

## 2 Methods

### 2.1 Study Subjects

The guppies used in this study were a gift of the Rodd Laboratory (University of Toronto) that were descendants of guppies collected from the ‘Houde’ tributary of the Paria river in Trinidad in 2008, supplemented with guppies collected from the same location in 2016. These fish had not been used in prior experiments or previously exposed to alarm cue. Like many low predation populations, major fish predators are absent from the Paria locale, but they do experience predation from freshwater prawns and *Anablepsoides hartii* (Reznick 1997). This predation regime has been suggested to result in low shoaling preferences in Paria guppies (Seghers 1974; Reznick 1997). Importantly, low predation guppies, including Paria guppies, respond to alarm cue, although their response differs from high predation guppies in magnitude and duration (Brown et al. 2010; Li et al. 2022).

Eight months prior to the study, we moved the fish to our laboratory at McGill University and housed them in large 150 L stock tanks fitted with a heater, a filter, gravel substrate and artificial plants. Tanks were maintained at 25°C ± 1°C and under a 12:12 light–dark cycle (lights on at 7:00 h). Each week, 30% water changes were done on each tank, and water pH, hardness, nitrites, nitrates and ammonia were measured. Fish were fed commercially available tropical fish flakes (TetraMin, Tetra, Melle, Germany) daily and supplemental decapsulated brine shrimp eggs (*Artemia* sp., Brine Shrimp Direct, Ogden, USA) three times a week. At the onset of the study, we collected newborn fry from these stock tanks of adult fish daily, and those born within 1 week of each other were randomly assigned to tanks in groups of five to nine in 20 L tanks. Tanks were then randomly assigned a cue (alarm cue or control). A total of 86 fry were allocated in this manner. This was done in a staggered manner such that groups of one control tank and one alarm cue tank were produced in batches each week until there were 12 tanks of fry, six for each treatment (see Table S1 for tank information and sample sizes at each step). These tanks were maintained under the same conditions as the stock tanks; however, water changes were reduced to once every 2 weeks to minimise any stress.

All procedures followed McGill University Animal Care and Use Committee Protocols (Protocol #7133/7708) and the guidelines from the Canadian Council on Animal Care and the Animal Behavior Society/Association for the Study of Animal Behaviour (ABS/ASAB).

### 2.2 Developmental Exposure

After a 5‐day acclimation period to the new tank, we began cue exposures. We exposed tanks to their assigned cue 3 days a week for 8 weeks (24 total cue exposures) on random days from Monday to Friday and between 9:00–17:00. Due to the staggered initiation of exposures, exposures began between October 2020 and February 2021. At the beginning of each day, we made fresh alarm cue (AC) following standard procedures (Brown and Godin 1999; Brown et al. 2009, 2010). Briefly, we homogenised skin extracts derived from mixed‐sex adult conspecifics from the Paria adult stock tank and then diluted with ddH2O to a concentration of 0.1 cm^2^ epithelial tissue/mL. After preparation, alarm cue was kept on ice and used within 1 h. Control cue (C) was made of ddH2O and kept on ice. 7 mL of assigned cue was administered to the top of the tank using a clean syringe and taking care not to disturb the fish in the tank. This amounted to a concentration of approximately 0.035 cm^2^ epithelial tissue/L in the 20 L tank, which is comparable to other studies (Brown and Godin 1999; Brown et al. 2010). It is difficult to quantify alarm cue exposure in the wild; however, we know that this concentration of alarm cue induces behavioural responses in guppies in the wild and that a high‐intensity predation environment likely involves multiple exposures to alarm cue a week (Brown and Godin 1999). After 8 weeks of cue exposures, we divided fish in each tank into sex‐specific 10 L tanks to mature for another 22 weeks without cue exposure and then, at an age approximately 210 days, we ran behavioural assays. This ensured fish were large enough for brain dissections.

### 2.3 Behavioural Assays and Data Analysis

After the 22‐week period without exposure to alarm cue, we presented each surviving fish (*n* = 81 total, 38 alarm cue fish and 41 control fish) with two behavioural assays in succession: a modified open‐field test and a shoaling test. Guppy behaviour has previously been found to be repeatable in both of these behavioural assays (Kniel et al. 2020). Assays were carried out between 9:00 and 17:00 from May to September 2021. Fish were not fed on the day of behavioural assays. Arena tanks were 20 L rectangular glass tanks with the sides covered in white corrugated plastic sheets to prevent reflections. We filled tanks with fresh conditioned and heated (25°C ± 1°C) water to 6 cm of depth and loosely scattered light‐coloured gravel along the bottom. For each assay, fish were allowed to habituate in the arena tank for 3 min in a transparent cylinder (diameter = 6 cm) placed at the centre of the tank. We then slowly lifted the cylinder to release the fish and begin the assay. The experimenter hid behind a barrier for the duration of the assay. The assays lasted for 5 min and were recorded using a 1080P HD Model N5 webcam (HDZIYU, Shenzhen, China) positioned 60 cm above the tank. In the modified open field test, a 10 cm × 10 cm artificial lawn aquarium plant that fish could hide in was placed in one corner of the tank (Figure S1). For 5 min, the fish was allowed to explore the tank or hide in the plant shelter. We used EthoVision XT v11.5 (Noldus et al. 2001) to quantify distance travelled (cm), time spent in the shelter (s), time spent in the outer edge of the tank (within the outer squares) (s), time spent moving (s) and time spent frozen (s). The last two measurements were only recorded while the fish was not in the shelter. Additionally, a virtual 4 × 8 grid was overlaid onto the arena video, and we extracted the amount of time a fish spent within each unique square. A fish had to spend at least 3 s within a square for it to count as ‘explored’. Immediately afterwards, we ran the shoaling test. Two identical glass cylinders with a 9 cm diameter were placed on each side of the tank—one empty and the other containing a shoal of four, unfamiliar adult females from the Paria population. The fish was then allowed to move around the tank monitored for 5 min. The side of the tank that contained the shoal container was alternated between every assay to control for any effect of tank side. One observer blind to cue treatment used BORIS v7.12.2 (Friard and Gamba 2016) to record time spent within four body lengths with each container (s). This is a commonly used measurement of shoaling (Chapman et al. 2008).

Data were analysed using R v4.3.2 (R Core Team 2022) statistical software. For the open field assay, we first assessed correlations between behavioural variables as these were measured in the same behavioural assay and have the potential to be highly correlated. Distance travelled and time spent moving assessed activity levels (Réale et al. 2007; Jacquin et al. 2016) whereas time spent in the outer edge of the tank, time spent frozen, and time in shelter were used to assess boldness (Jacquin et al. 2016; Jolles et al. 2019). Time spent moving, time spent frozen and distance travelled were highly correlated (*r* > 0.7). Therefore, we dropped time spent frozen and time spent moving but retained distance travelled as a proxy for activity. We added together the time spent in the shelter and the time spent in the outer edge as a proxy for boldness. This was necessary as a fish that spent more time in the refuge (indicative of shyer behaviour) would likely have a lower time outer edge score, due to less time being in the arena to be scored for time in the outer edge, resulting in conflicting boldness scoring. Lastly, we used unique squares explored as a proxy for exploration (Cattelan et al. 2020). None of our retained behavioural proxies were highly correlated (*r* < 0.33). For the shoaling test data, we used preference for the container with the shoal as a behavioural proxy for shoaling. This was calculated by subtracting time spent shoaling with the empty container from time spent shoaling with the shoal container. We ran models with the *lme4* package (Bates et al. 2015) using each behavioural proxy as the outcome variable in separate models. For squares explored, we used a generalised mixed model with a Poisson distribution as it was count data. For the rest of the behavioural proxies, we used linear mixed models with restricted maximum likelihood. Some previous behavioural studies in guppies have found an impact of sex and body mass on activity, boldness, M and exploratory behaviour (Harris et al. 2010; Santostefano et al. 2019) and sex on shoaling (Griffiths and Magurran 1998). Therefore, for the open field test models we included sex, cue, and mass and for the shoaling model we included sex and cue as predictors along with their interactions. We additionally assessed mass in the shoaling model but it was not significant (*p* = 0.928), so we removed it. Initial t tests confirmed that alarm cue had no impact on fish mass for either sex (Females: *t* = −0.22, df = 43.38, *p* = 0.82; Males: *t* = 1.59, df = 27.26, *p* = 0.12). We also included home tank as a random effect to control for any tank effects in all models. For models where interactions between terms were not significant (*p* > 0.05), we re‐ran models without interactions and present these results only. We verified assumptions of our mixed models using the *DHARMa* R package (Hartig 2022). Using the *car* R package (Fox and Weisberg 2019), we calculated Chi‐square and *p* statistics for each model. We used the *r2glmm* package (Jaeger et al. 2017) to calculate model *R* ^2^ and semi‐partial *R* ^2^ for each fixed effect using the Nakagawa and Schielzeth approach (Nakagawa and Schielzeth 2013). Lastly, we checked for a significant preference for the container containing the shoal within all treatment group and sex combinations (AC females, C females, AC males and C males) using paired t tests.

### 2.4 DNA Extraction and Whole‐Genome Bisulfite Sequencing

Immediately following behavioural assays, we euthanized fish by immersion in ice water. Within 3 min, we measured fish mass and length and dissected out brains. Brains were stored in RNAlater (Thermofisher Scientific, Waltham, USA) at 4°C and then frozen at −80°C within 24 h. We extracted DNA from whole brains using AllPrep DNA/RNA Kits (Qiagen, Hilden, Germany) following the manufacturer's protocol. In total, brain samples from 76 fish had enough DNA for sequencing (35/38 alarm cue fish and 40/43 control cue fish). Whole genome bisulfite sequencing (WGBS) library preparation and sequencing was carried out at the McGill Genome Center (Montréal, Canada). Paired‐end libraries of 150 bp were prepared for each fish and sequenced on the Illumina NovaSeq6000 S4 (Illumina, San Diego, United States) along with guppy samples for a different project, with 69 individuals pooled per lane.

### 2.5 WGBS Data Processing

We processed sequence reads using the nf‐core/methylseq pipeline v1.6.1 (Ewels et al. 2019, 2020). This pipeline uses FASTQC v0.11.9 (Andrews 2019) to analyse raw reads and Trim Galore! v0.6.5 (https://www.bioinformatics.babraham.ac.uk/projects/trim_galore/) to trim adaptor sequences and low‐quality reads. The Bismark v0.22.3 (Krueger and Andrews 2011) pathway in the pipeline was used to align reads to the guppy reference genome (GenBank assembly accession GCA_000633615.2) with BowTie2 v2.5.0 (Langmead and Salzberg 2012) and extract methylation data. The average mapping efficiency was 64.28% ± 0.90% (Table S2). MultiQC (Ewels et al. 2016) is used in the pipeline to generate alignment reports across all samples. After analysing quality check reports, one sample, DAC4M1, was removed from further analysis due to low coverage (mean coverage = 2.3×). Only CpG context methylation was analysed; however, we also quantified methylation at non‐CpG sites and found that an average of 0.833% ± 0.052% of CHG cytosines and 0.940% ± 0.059% of CHH cytosines were methylated, suggesting a highly efficient bisulfite conversion. The percent methylated CpGs was highly similar between control fish (74.72% ± 0.336%) and alarm cue‐exposed fish (74.697% ± 0.237%).

### 2.6 Identification of Differentially Methylated Sites and Regions

Prior to methylation analysis, we merged coverage and methylation levels from both strands using a custom Python script (https://github.com/rcristofari/penguin‐tools/blob/master/merge_CpG.py). Differential methylation was analysed using the *methylKit* package v1.18.0 (Akalin et al. 2012). We filtered CpG sites to a minimum of five reads in at least 60% of fish per treatment group and removed sites that were in the 99.9th percentile of coverage to control for PCR bias and sites that had low variation defined as a percent methylation standard deviation less than 2. We then median‐normalised coverage values between samples. Single nucleotide polymorphisms (SNPs) can result in incorrect methylation calls if C‐to‐T or G‐to‐A SNPs are falsely interpreted as unmethylated cytosines and, therefore, should be corrected for. We identified SNPs across all samples using BS‐SNPer (Gao et al. 2015) with the following quality filters: minimum base quality of 15, minimum coverage 10, maximum coverage of 1000, minimum read mapping value of 20, minimum mutation rate of 0.02, minimum mutation reads number of 2, threshold frequency for calling heterozygous SNPs of 0.1 and threshold frequency for calling homozygous SNPs of 0.85. Then, we isolated C to T SNPs and used the *GenomicRanges* package (Lawrence et al. 2013) to remove them from further analysis. We identified 5,892,571 SNPs, of which 832,862 were C to T SNPs. Our filtering resulted in a dataset of 9,028,900 CpG sites for females and 9,343,839 for males.

Using *methylKit*, we detected differentially methylated sites (DMSs) and regions (DMRs) for each sex separately through logistic regressions for each CpG site, with tank as a covariate. Significance was evaluated using a chi‐square test and the sliding linear model (SLIM) method, yielding *q*‐values. We deemed sites and regions as significant if they exhibited a minimum of 20% differential fractional methylation between fish exposed to alarm cues and those exposed to control cues, with *q*‐values < 0.0125 (Akalin et al. 2012; Heckwolf et al. 2020). For DMR identification, a tiling method was employed with a sliding window size of 100 bases and a step size of 100 bases. CpGs were initially filtered to a minimum of three reads, and then each region was subsequently filtered to a minimum of five reads after tiling. A chi‐square test was used to determine if there was a higher proportion of significant DMSs and DMRs in males or females. The differential methylation analysis method we employed can lead to elevated levels of false positives (Wreczycka et al. 2017). Therefore, we validated our results by rerunning the DMS analysis for each sex using the same parameters but with overdispersion correction, which greatly reduces sensitivity but increases specificity (Wreczycka et al. 2017).

We ran hierarchical clustering with Euclidean distance and Ward's linkage using the *cluster* v2.1.4 package (Maechler et al. 2022) on DMSs and DMRs. Additionally, a chi‐square goodness of fit test was used to determine if DMSs and DMRs were significantly more hypo‐ or hypermethylated. The direction of methylation was determined by comparing alarm cue fish against control fish such that hypermethylation means there is more methylation in the alarm cue fish.

### 2.7 Association of Methylation With Behavioural Data

We used elastic net regressions implemented in the *glmnet* v4.1–8 package (Tay et al. 2023) to identify DMSs that may be associated with behaviour. Elastic net regressions use the penalties from both lasso and ridge approaches to allow some regression coefficients to go to zero, which yields a type of feature selection that is ideal for datasets with multicollinearity and high dimensionality such as in methylation datasets (Zou and Hastie 2005). We focussed on shoaling as this is the only behaviour that was significantly impacted by developmental cue exposures. Since DMSs were sex‐specific, we ran a separate elastic net model for each sex. Preference for shoal was the dependent variable, and methylation data from the DMSs were the predictor variables. Prior to model training, all data were scaled and centered. The *caret* v6.0–94 package (Kuhn 2008) was used to train models on the training data and identify the optimum alpha and lambda values using 10‐fold cross validation repeated 10 times. In 10‐fold cross validation, the data are shuffled and split into 10 equal parts, with the training data being ninefold (so 90% of the data), and the remaining onefold (10%) is used as the test. Final models were selected using the lambda value within one standard error of the minimum, and *R* ^2^ and root‐mean‐square error (RMSE) were calculated. We used the magnitude of the absolute values of the regression coefficients estimated by the elastic net models to determine which DMSs had the strongest effects on behaviour. To further investigate these strong effect DMSs in the absence of weaker effect DMSs that could negatively impact models, we ran linear mixed models with the top 10 non‐zero DMSs (with regards to regression coefficients) and cue treatment as fixed effects, shoaling preference as the dependent variable, and tank as a random effect. The cut‐off of the top 10 DMS was chosen as regression coefficients decreased rapidly in magnitude around this cut‐off. Models were assessed for assumptions and significance in the same way as behavioural models outlined above (Section 2.3).

### 2.8 Functional Annotation and Gene Ontology Analysis

We identified the genomic feature for each DMS, DMR, and CpG that passed filtering using the ENSEMBL guppy database (release 108; accessed Feb 2023) and the *genomation* R package v1.35.0 (Akalin et al. 2015). For overlapping features, we gave precedence to promoters > exons > introns > intergenic regions (Akalin et al. 2012), with the promoter region defined as 1500 bp upstream and 500 bp downstream from the transcription start site (TSS). To assess if there were shifts in the distribution of DMSs and DMRs, we compared the distributions of all DMSs to a null distribution based on the distribution of all CpG sites using a G test. Post hoc G tests were used to identify which features deviated significantly from the null distribution. The Hommel method (Hommel 1988) was used to adjust for multiple testing.

To identify the nearest transcription start site (TSS) to a DMS or DMR, we used the *GenomicRanges* R package (Lawrence et al. 2013). A gene was considered differentially methylated if a DMS or DMR was within 10 kb of the TSS. The R packages *GOstats* (Falcon and Gentleman 2007) and *GSEABase* (Morgan et al. 2023) were used to uncover over‐represented biological processes for hypermethylated and hypomethylated genes. A conditional hypergeometric gene ontology (GO) term enrichment analysis was performed with all genes associated with any CpG site retained after filtering as the background gene set. We corrected *p*‐values for multiple testing using a false discovery rate and considered false discovery rate‐corrected *p* <= 0.05 as the significance threshold.

## 3 Results

### 3.1 Effect of Alarm Cue Exposure on Behaviour

**FIGURE 1:**
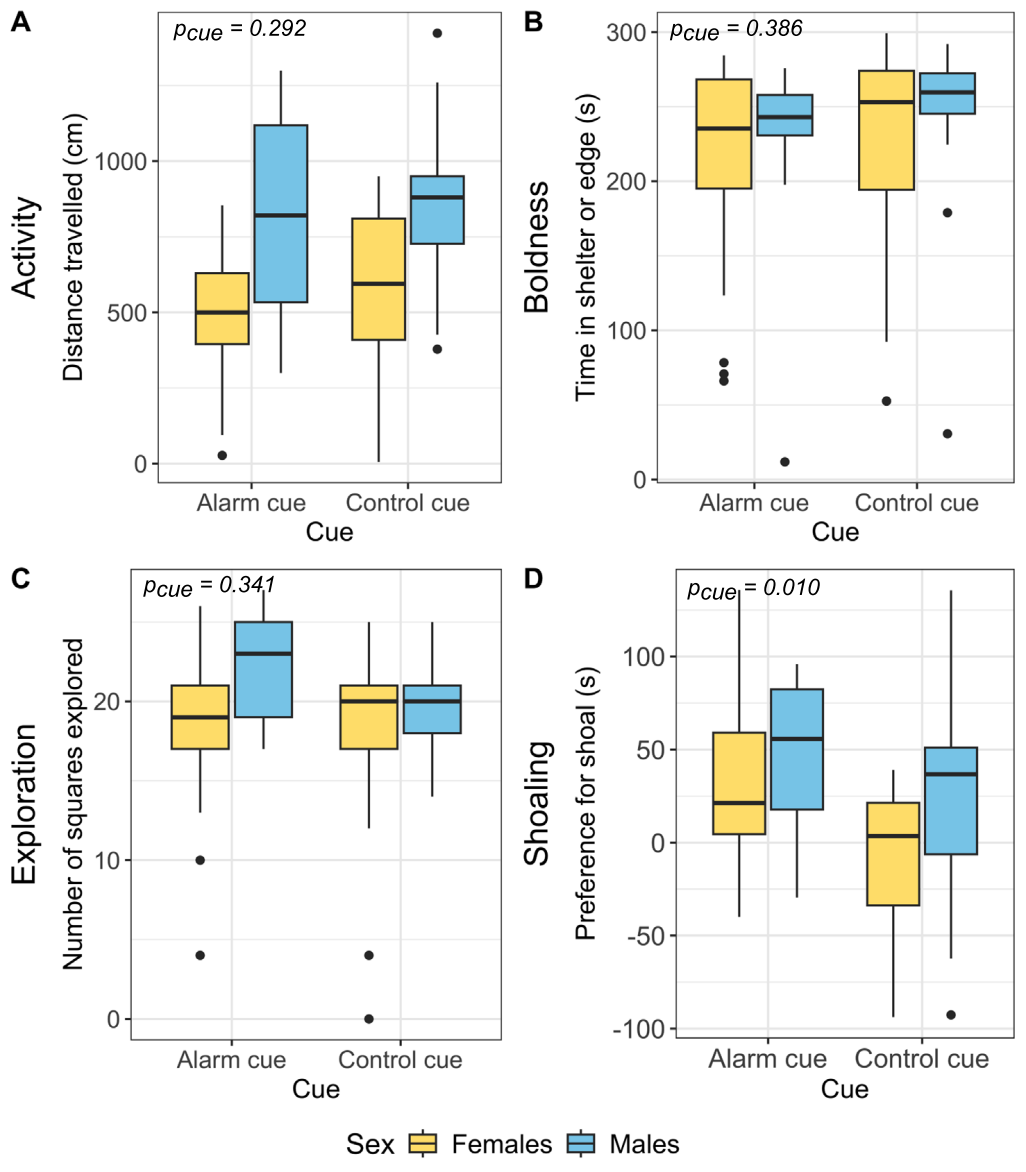
Behavioural measurements for alarm cue‐ and control cue‐exposed guppies. (A) Distance travelled (cm) (proxy for activity), (B) time spent in shelter and in outer edge of tank (s) (proxy for boldness) and (C) number of squares explored (proxy for exploration) were measured in a modified open field test. (D) Preference for a container containing a female shoal over an empty container (proxy for shoaling) was measured in a shoaling test. A positive number indicates a preference for the container containing a shoal. Linear mixed models were run with each behavioural measurement as the outcome and cue type as a predictor. Tank was included as a random effect in all models. Sex was included as a fixed effect in all models, and body mass was included as a fixed effect in all models except for the shoaling one. Significance of cue in the linear mixed models is shown on each plot.

Cue treatment had no significant impact on measures of activity (distance travelled), exploration (unique squares explored), or boldness (time spent in shelter and time spent in outer edge) (Table 1; Table S3; Figure 1A–C). While sex and body mass alone did not have significant effects on activity, the interaction between sex and mass was significant such that for males but not for females, as mass increased so did the distance travelled. Sex also had a significant impact on exploration with males entering more squares than females. Sex, body mass, and the interaction between the two had no significant effects on boldness. The model *R* ^2^ was 0.307 for the activity model, 0.067 for the exploration model, and 0.040 for the boldness model. All models had low (< 0.1) semi‐partial *R* ^2^ for all variables (Table S3). Cue significantly impacted shoaling such that fish exposed to alarm cue as juveniles shoaled more than control cue‐exposed fish (Figure 1C). Additionally, males shoaled more than females. No interactions were significant in the shoaling model. The model *R* ^2^ was 0.148 with semi‐partial *R* ^2^ of 0.116 and 0.063 for cue and sex, respectively. We found that in all treatment and sex combinations, there was a significant preference for the container with the shoal over the empty container, except for the control females (AC females: *t* = 3.781, df = 24, *p* = 0.0009; C females: *t* = −0.920, df = 21, *p* = 0.368; AC males: *t* = 3.726, df = 12, *p* = 0.003; C males: 1 = 2.288, df = 20, *p* = 0.033).

**TABLE 1:** Results of linear mixed models on behavioural measurements.

	*X* ^2^	df	*p*
*Activity (distance travelled)*			
Intercept	13.652	1	**0.0002**
Cue	1.107	1	0.292
Sex	0.006	1	0.939
Mass	0.170	1	0.680
Sex × Mass	4.617	1	**0.032**
*Boldness (Time in shelter or frozen)*			
Intercept	43.225	1	< 0.0001
Cue	0.752	1	0.386
Sex	2.204	1	0.138
Mass	0.544	1	0.461
*Exploration (Squares explored)*			
Intercept	675.450	1	**< 0.0001**
Cue	0.903	1	0.341
Sex	5.319	1	**0.021**
Mass	0.783	1	0.378
*Shoaling (Preference for shoal)*			
Intercept	9.898	1	**0.002**
Cue	6.713	1	**0.010**
Sex	5.751	1	**0.016**

*Note:* Significant *p*‐values are bolded (*p* < 0.05). Tank was included as a random effect in all models. *n* = 81, 38 alarm cue fish and 41 control fish.

### 3.2 Differential Methylation Analysis

We identified 8769 DMSs and 51 DMRs for females and 27,916 DMSs and 402 DMRs for males. Of these, 638 DMSs and 7 DMRs overlapped between the sexes. There were significantly more significant DMSs (*X* ^2^ = 9368.20, df = 1, *p*‐value < 0.0001) and DMRs (*X* ^2^ = 269.75, df = 1, *p*‐value < 0.0001) in males than in females. Given our methylation cut‐off of 20%, methylation differences in DMSs ranged from 20% to 58.57% for females and 20% to 80.23% for males. Methylation differences in DMRs ranged from 20% to 30.17% for females and 20% to 58.25% for males. The observed sex difference in the number of DMSs and DMRs could be due to differing levels of inter‐individual methylation variability. If females have more methylation variability than males, this could make it more difficult to detect differential methylation. To examine this, we used standard deviation as a measurement of methylation variability and calculated this for every CpG in both sexes. We then compared the mean standard deviation between females and males using a t test to detect significant differences in variability across all CpG sites. There was a significant difference in the mean standard deviation, but males had a significantly larger mean than females (*t* = −13.331, df = 2,634,031, *p* < 0.0001), and the difference in average standard deviations was quite small (Females = 10.141, Males = 10.240). Therefore, we do not suspect that sex differences in methylation individual variability contributed greatly to our findings. While we identified fewer DMSs in our validation analysis using overdispersion correction, as expected given the greatly reduced sensitivity of this technique, there were still significant DMSs for both sexes (Females: 38; Males: 646) and we observed similar patterns of sex differences.

**FIGURE 2:**
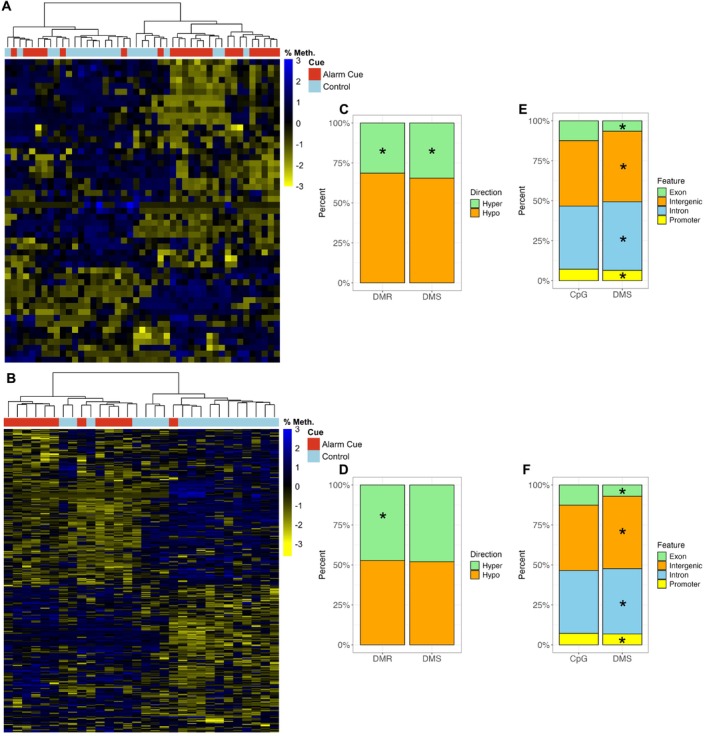
Differential methylation analysis results. (A and B) Heatmap of differentially methylated regions (DMR) with hierarchical clustering of samples for (A) females and (B) males. Each row is a DMR and each column is an individual. Scaled percent methylation for each DMR is displayed in the heatmap. (C and D) Proportion of DMSs and differentially methylated regions (DMRs) that are hypo‐ or hyper‐methylated for (C) females and (D) males. Direction of methylation is determined by comparing alarm cue fish to control fish such that hypermethylation means there is more methylation in the alarm cue fish. Asterisk indicates a significant difference between the proportion of hyper‐ and hypo‐methylation found using a Chi‐Square goodness‐of‐fit test (*p* < 0.0001). (E and F) Proportion of CpGs and DMSs, located in exons, intergenic regions, introns or promoters for (E) females and (F) males. Asterisks indicate significant differences from the null distribution (constructed from the distribution of all CpGs) found using a G‐test (*p* < 0.05 for all).

We found that samples clustered largely by cue, but there was significant mixing between the alarm cue and control fish, especially for females (DMRs: Figure 2A,B, DMSs: Figure S1). There were more hypomethylated than hypermethylated DMSs and DMRs for both sexes; however, this difference was only significant for DMSs in males (DMSs: *X* ^2^ = 43.66, df = 1, *p* < 0.0001; DMRs: *X* ^2^ = 1.20, df = 1, *p =* 0.13), while it was significant for both DMSs and DMRs in females (DMSs: *X* ^2^ = 846.8, df = 1, *p* < 0.0001; DMRs: *X* ^2^ = 7.08, df = 1, *p =* 0.008) (Figure 2C,D).

### 3.3 Association Between Methylation and Shoaling

The elastic net regression for females had low predictive power and high root‐mean‐squared error (RMSE) (*R* ^2^ = 0.290 ± 0.283, RMSE = 42.738 ± 16.515) indicating low performance. The linear mixed model using the top 10 DMSs had an *R* ^2^ of 0.608, and two DMSs were statistically significant (*p* = 0.004 and 0.046) with effect sizes of 0.154 and 0.077 (Table S4, Figure S3A,B). For males, the elastic net model had higher predictive power but still high RMSE (*R* ^2^ = 0.633 ± 0.383, RMSE = 47.749 ± 19.470). The linear mixed model for males had an *R* ^2^ of 0.726, and one DMS was statistically significant (*p* = 0.027) with an effect size of 0.166 (Table S4, Figure S3C).

### 3.4 Gene Ontology Enrichment Analysis

The distribution of DMSs differed from the null distribution for both sexes (Figure 2E,F, Table S5 for full results). For females and males, DMSs showed a significant increase in introns and intergenic regions and a decrease in promoters and exons. DMSs did not obviously appear on specific chromosomes in either sex (Figure S4). In females, DMSs that were hypermethylated were involved in various cellular responses and metabolic processes, and in males, they were involved in signalling, regulation of cellular and biological processes, and cell communication (Figure S5). Hypermethylated DMRs were similarly involved in various metabolic processes for males and females, but also hormonal regulation in females (Figure S6). Hypomethylated DMSs were involved in behaviour, vasoconstriction and multicellular organismal process in females, whereas in males, they were involved in the regulation of synaptic signalling and tissue morphogenesis (Figure S7). Hypomethylated DMRs were involved in responses to stimulus, immune responses and neuropeptide signalling pathways in females, and in vasoconstriction and regulation of cyclase activity in males (Figure S8). See Data S1 for full GO enrichment results.

## 4 Discussion

DNA methylation (DNAm) has recently emerged as a potential mechanism underlying developmental behavioural plasticity. We used the well‐studied guppy system to investigate whether early‐life exposure to an ecologically relevant cue, predation stress, had lasting impacts on behaviour and DNAm. Fish exposed to alarm cue early in life shoaled more than fish exposed to a control cue. Moreover, we observed shifts in neural DNAm in genes potentially related to behavioural regulation. Methylation at several of these differentially methylated sites was significantly associated with individual differences in shoaling, suggesting that DNAm could be a molecular mechanism responsible for this developmental behavioural plasticity. Additionally, males and females differed in the magnitude of their DNAm responses, implying that DNAm could be playing distinct roles in each sex.

### 4.1 Early‐Life Predation Stress Induces Shifts in DNAm in Adulthood

Early‐life predation stress induced shifts in DNAm into adulthood. Our results add to a growing body of evidence showing that DNAm is environmentally responsive to a variety of environmental cues including temperature (Fellous et al. 2022), salinity (Heckwolf et al. 2020), and nutritional stress (Sepers et al. 2021) and thus provide support for the idea that DNAm could be a mechanism of environmentally directed phenotypic plasticity (Bossdorf et al. 2010; Putnam et al. 2016). DNAm could play an especially important role in regulating behavioural plasticity due to the environmentally responsive nature of behavioural traits. Early‐life stress has provided a good paradigm for investigating this role, but most studies have been done in rats or mice thus far (Murgatroyd et al. 2009; Anier et al. 2014; Catale et al. 2020). Two previously mentioned studies point to DNAm playing a role in behavioural plasticity in fish (McGhee and Bell 2014; Fellous et al. 2018). However, one study did not measure methylation directly (McGhee and Bell 2014) and the other used differences in structural environment (Berbel‐Filho et al. 2020). We build on these studies by investigating early‐life exposure to predation, which is a widespread, ecologically relevant stressor that leads to phenotypic shifts that could have adaptive impacts. Previous studies in mice suggested a role of DNAm in behavioural responses to predation (Bowen et al. 2014; Kigar et al. 2017) but they do not consider a developmental timescale or measure methylation across the whole genome as we do here. If the shifts in DNAm that we observed are directly linked to adaptive shifts in shoaling, then DNAm would have adaptive consequences. However, direct measures of adaptive effects would be needed to confirm this.

Ties between methylation and phenotypes have not always been easy to decipher due to complex interactions between DNAm, gene expression and phenotypes (Jones 2012). Because DNAm can play a critical role in gene expression (Jones 2012; Wagner et al. 2014; Anastasiadi et al. 2018), it is possible that the changes in DNAm we uncovered lead to shifts in gene expression. We observed a decrease of DMSs in promoters and exons and an increase in introns and intergenic regions, which could suggest that these shifts in DNAm are indeed playing a gene regulatory role. Typically, decreased methylation, especially in promoters, has been associated with increases in gene expression, but this is not always the case, as DNAm can also lead to alternative splicing patterns or even decreases in gene expression (Jones 2012; Maor et al. 2015; Shayevitch et al. 2018). Therefore, our gene ontology (GO) results should be interpreted with these limitations in mind. Still, we found that hyper‐ and hypomethylated DMSs and DMRs were in genes that could play a variety of roles in the developmental behavioural plasticity we observed. For example, females had many over‐represented GO terms involved in hormonal regulation. Hormones play a critical role in the expression of behaviours, with cortisol being especially important for regulating stress responses and responses to predation in fish (Barreto et al. 2014). Males had over‐represented GO terms involved in signalling, cell communication and chemical synaptic transmission, which could indicate shifts in the neural circuitry involved in responding to predation threats (Pereira and Moita 2016). These over‐represented genes could be further investigated to determine what functional impacts DNAm has on their expression and behaviour.

Another way to infer the connection between methylation and phenotypes is through finding associations between specific DMSs and traits. Analysing data with such a high ratio of variables (DMSs) to samples (number of fish) is challenging. Machine learning techniques, such as the elastic net regression we employed, can use regularisation methods that aim to reduce overfitting of models by limiting analysis to important variables only (Zou and Hastie 2005). However, these methods have a focus on prediction, not hypothesis testing, making them difficult to interpret in the current context. Therefore, these results should be interpreted with caution. We found that methylation across all DMSs did not predict shoaling well in the elastic models, which suggests that overall methylation patterns cannot predict this behaviour. This finding could be due to the many other unaccounted factors that modify behaviour through the complex interactions between DNAm, gene expression, and phenotypes or the many DMSs included in the model that likely have no impact on behaviour. However, we also used the elastic net models to select variables to include in linear mixed models. This approach yielded significant associations between methylation and behaviour. In females, two DMSs had significant impacts on shoaling and in males one DMS did. This provides evidence consistent with a role of DNAm in developmental behavioural plasticity. While behavioural traits are often considered polygenic (Bubac et al. 2020), it is possible that methylation in a few specific regions could have strong functional impacts. Further research should simultaneously analyse DNAm, gene expression, and phenotypic datasets, which could better uncover the functional impact of DNAm on developmental behavioural plasticity.

### 4.2 Early‐Life Predation Stress Induces Developmental Behavioural Plasticity

We found that exposure to early‐life alarm cue, simulating high predation risk, induced a developmentally plastic shift in shoaling in guppies. The guppy population we studied typically shows low shoaling tendencies (Magurran and Seghers 1991), as we found in females exposed to control cue, yet shoaling was increased by early‐life exposure to alarm cue. Other studies have shown increases in anti‐predator behaviour in response to early‐life predation stress, such as in the European minnow ( *Phoxinus phoxinus* ) (Magurran 1990b) and tadpoles ( *Rana lessonae* and *Rana esculenta* ) (Semlitsch and Reyer 1992). In the same population of guppies studied here, exposure to visual and olfactory cues of a predator, *Anablepsoides hartii*, during early development increased shoaling but only when exposed to a current predation risk (Li et al. 2022). This differs from our finding that early‐life predation stress increased shoaling even in the absence of a current cue. These differences could be due to varying perception of predation risk under alarm cue exposures versus exposures to cues from *A. hartii* which are considered a low‐intensity predator that mostly feeds on juveniles (Reznick et al. 1997). Testing under current predation risk could have shown an even stronger difference between control and alarm cue‐exposed fish. In nature, guppies from high predation populations show increased shoaling compared to low predation populations and this has a genetic basis, suggesting increased shoaling is an evolved response to predation that likely has adaptive benefits (Seghers 1974; Magurran and Seghers 1991; Huizinga et al. 2009). While guppy predators are known to attack larger groups of guppies more than smaller groups, guppies in a shoal have higher chances of surviving predator attacks (Krause and Godin 1995; Li et al. 2022). Developmentally plastic behavioural responses may be more likely to occur for adaptive behaviours due to past selection.

Exposure to early‐life predation stress did not induce shifts in activity, exploration, or boldness. Previous studies have found that high predation guppies are bolder (Harris et al. 2010) and less exploratory (Burns et al. 2016) but the extent to which these behaviours are developmentally plastic remains unclear. One study found that guppies exposed to predator cues during development increased time spent swimming and time spent in the inner circle of a tank, both of which could be described as measurements of boldness (i.e., less time frozen, less time at the edge of the tank) (Stein and Hoke 2022). However, our conflicting findings could be due to the different way that we measured boldness in the open field test (i.e., the addition of a shelter). Other intrinsic, individual traits may also impact the expression of these behaviours more strongly than environmental cues (discussed further below).

### 4.3 Sex Differences in Behaviour and DNAm Responses

The magnitude of DNAm response differed greatly between males and females. Males showed many more DMSs and DMRs than females, suggesting that males could be more responsive than females to developmental alarm cue exposures. As DMSs did not obviously appear to be grouped on specific chromosomes, including the sex chromosomes, we do not expect that sex chromosomes contributed to these sex differences. DNAm could play a different role in developmental plasticity or could be involved in different behavioural responses in males and females. Males exhibit developmental plasticity in response to predation stress in their mating behaviour, which is also associated with differences in brain size and morphology (Yang et al. 2023). We did not measure mating behaviour, so it is unknown whether our fish also displayed developmental plasticity in these traits. As such, potential changes in unmeasured mating behaviours could explain why males show more DNAm shifts than females. Additionally, males adjust their behaviour based on social cues of females (Evans et al. 2002). If males are responding to social cues from females rather than directly to alarm cues, differences in the brain networks involved in processing social information (O'Connell and Hofmann 2012) could explain why there is little overlap in DMSs and DMRs between the sexes. Future studies could investigate whether DNAm underlies developmental plasticity in mating behaviour and the processing of social cues in males.

Sex and body mass had very low or no impact on activity, exploration and boldness. Studies have frequently found no or a very small impact of mass and sex on these behaviours in guppies (Harris et al. 2010; Kemp et al. 2022). We also found that males shoaled more than females. However, this could be due to our use of an all‐female shoal and the male's incentive to engage in mating attempts.

### 4.4 Future Directions

This research opens several avenues for future research. One limitation of our study is that we used whole brain tissue, comprised of many different cell types and brain regions. Changes in DNAm are likely not homogeneously distributed across the brain, and uncovering which specific regions or cells show shifts in DNAm could provide more information on its function. Future studies could use laser capture microdissection to select specific cells (Datta et al. 2015); however, this would be a significant undertaking. Newly developed spatial sequencing technologies may also be useful for this (Zhang et al. 2023). Additionally, our findings are correlative, and direct manipulation of DNAm would be required to confirm the relationship between DNAm and behaviour. Without manipulative studies, we cannot establish whether alarm cue induced shifts in DNAm that then resulted in shifts in behaviour or if instead the alarm cue induced shifts in behaviour that then resulted in shifts in DNAm. DNA methyltransferase inhibitors can alter the level of methylation in the brain and could be useful for manipulative studies (Miller et al. 2008); however, their effects are widespread and may not be targeted enough. A modification of the CRISPR/Cas9 system for introducing site‐specific methylation changes may prove useful (McDonald et al. 2016). Additionally, we employed *methylKit* to analyse differential methylation, which is known to lead to higher levels of false positives but offers the highest level of sensitivity, something essential for our study that had a lower sequencing depth (Wreczycka et al. 2017). While *methylKit* represented the best option for us (and is still considered to be among the most accurate options), further studies using deeper sequencing and alternative methylation analysis methods could reveal more insight. Lastly, there may be individual differences in DNAm responses to early‐life stress that could have important phenotypic effects, potentially leading to a genotype × environment effect that is mediated by the genome. Future studies should investigate whether genotypes vary in their epigenomic responses and how this could impact developmental behavioural plasticity.

### 4.5 Conclusions

Recent evidence has suggested that DNAm could play a role in developmental behavioural plasticity in response to early life stress, but our current understanding remains limited. In this study, we showed that exposure to alarm cue throughout early life, inducing predation stress, had lasting impacts on shoaling behaviour and DNAm in Trinidadian guppies. Shifts in DNAm occurred in many genes involved in behavioural regulation, and shifts in DNAm at specific sites were associated with differences in shoaling. These results suggest that DNAm could underlie developmental behavioural plasticity in anti‐predator behaviours in guppies. We also found important sex differences in DNAm responses that could indicate sex differences in the mechanisms of predator‐induced developmental plasticity that warrant further investigation. Future studies that work to uncover the relationship between DNAm and behavioural phenotypes will be important to determine the molecular mechanisms of behavioural plasticity and the factors that contribute to behavioural variation.

## Author Contributions

J.A.F. and M.F.G. conceptualised the study design. J.A.F. carried out laboratory work, analysed data and wrote manuscript. S.M.R., R.D.H.B. and M.F.G. gave feedback on design and provided funding/facilities. All authors gave feedback on the paper.

## Conflicts of Interest

The authors declare no conflicts of interest.

## Supporting information


Data S1.

## Data Availability

The WGBS data presented in this study are deposited in the NCBI Sequence Read Archive (SRA) repository (BioProject ID: PRJNA1220133). Behavioural data and all code can be found on Dryad (doi:10.5061/dryad.dbrv15fch). Analysis code can also be found on GitHub (https://github.com/janayfox/Guppy‐Cue‐Exposure).
