# Supplementary material for: Developmental Behavioural Plasticity and DNA Methylation Patterns in Response to Predation Stress in Trinidadian Guppies
Source: Mol Ecol. 2025 Jun 13;34(14):e17831. doi: 10.1111/mec.17831 (PMC12237088; doi:10.1111/mec.17831)
Supplement: Supplementary file 1 — Data S1. [file MEC-34-e17831-s001.docx]

**Supplemental Information for:**

**Developmental behavioural plasticity and DNA methylation patterns in response to predation stress in Trinidadian guppies**

Janay A. Fox, Simon M. Reader, Mélanie F. Guigueno, Rowan D. H. Barrett

**Table of Contents:**

| Supplemental Table 1. Information on the tanks used for developmental cue exposures. | Page 2 |
| --- | --- |
| Supplemental Table 2. Read counts and alignment statistics for all samples. | Page 2 - 4 |
| Supplemental Table 3. Results of linear mixed models of effect of alarm cue versus control cue on behavioural measurements. | Page 4 - 5 |
| Supplemental Table 4. Full linear mixed model results for associations between differentially methylated regions (DMSs) and shoaling. | Page 5 - 6 |
| Supplemental Table 5. G test results for the proportion of differentially methylated sites (DMSs) and regions (DMRs) distributed in genomic features compared to a null distribution. | Page 6 |
| Supplemental Figure 1. Schematics of behavioural assays, plan views. | Page 7 |
| Supplemental Figure 2. Heatmaps of differentially methylated sites (DMS) for (A) females and (B) males. | Page 8 |
| Supplemental Figure 3. Linear mixed models showing association between percent methylation at specific differentially methylated regions (DMRs) and shoaling in (A) females and (B to D) males. | Page 9 |
| Supplemental Figure 4. Manhattan plots of differentially methylated sites (DMS) for (A) females and (B) males. | Page 10 |
| Supplemental Figure 5. Gene ontology enrichment analysis results for hypermethylated differentially methylated sites (DMS) for females and males. | Page 11 |
| Supplemental Figure 6. Gene ontology enrichment analysis results for hypermethylated differentially methylated regions (DMR) for females and males. | Page 12 |
| Supplemental Figure 7. Gene ontology enrichment analysis results for hypomethylated differentially methylated regions (DMS) for females and males. | Page 13 |
| Supplemental Figure 8. Gene ontology enrichment analysis results for hypomethylated differentially methylated regions (DMR) for females and males. | Page 14 |

**Supplemental Table 1. Information on the tanks used for developmental cue exposures.**

| **Tank** | **Cue** | **Ind.** | **Fem.** | **Mal.** | **Deaths** | **Cue Exp.**  **Start** | **Behav. Assay**  **Date** | **Fem.**  **Seq.** | **Mal.**  **Seq.** |
| --- | --- | --- | --- | --- | --- | --- | --- | --- | --- |
| AC2 | AC | 5 | 4 | 0 | 1 | 15/10/2020 | 14/05/2021 | 3 | 0 |
| C2 | C | 5 | 2 | 2 | 1 | 16/10/2020 | 15/05/2021 | 2 | 2 |
| AC3 | AC | 5 | 2 | 2 | 1 | 01/11/2020 | 28/05/2021 | 2 | 2 |
| C3 | C | 7 | 3 | 4 | 0 | 02/11/2020 | 29/05/2021 | 3 | 3 |
| AC4 | AC | 7 | 5 | 1 | 1 | 06/01/2021 | 07/08/2021 | 5 | 0 |
| C4 | C | 8 | 5 | 3 | 0 | 07/01/2021 | 08/08/2021 | 5 | 2 |
| AC5 | AC | 9 | 5 | 4 | 0 | 27/01/2021 | 25/08/2021 | 4 | 4 |
| C5 | C | 8 | 5 | 3 | 0 | 28/01/2021 | 26/08/2021 | 5 | 3 |
| AC6 | AC | 7 | 3 | 3 | 1 | 09/02/2021 | 06/09/2021 | 3 | 3 |
| C6 | C | 8 | 4 | 4 | 0 | 10/02/2021 | 07/09/2021 | 4 | 3 |
| AC7 | AC | 9 | 6 | 3 | 0 | 25/02/2021 | 23/09/2021 | 6 | 3 |
| C7 | C | 8 | 3 | 5 | 0 | 27/02/2021 | 25/09/2021 | 3 | 5 |

AC = alarm cue, C = control, Ind. = Total number of individuals in tank at beginning of study, Fem. = number of females in tank, Mal. = number of males in tank, Cue Exp. Start = date that cue exposures started, Behav. Assay Date = date that behavioural assays were carried out, Fem. Seq. = number of females sequenced from that tank, Mal. Seq. = number of males sequenced from that tank. Some individuals not sequenced due to poor DNA extractions.

**Supplemental Table 2. Read counts and alignment statistics for all samples.**

| **ID** | **Tank** | **Treatment** | **Reads** | **Uniquely Mapped** | **Mapping Efficiency (%)** | **No. CpGs After Alignment** |
| --- | --- | --- | --- | --- | --- | --- |
| DAC2F4 | AC2 | AC | 50888569 | 33154510 | 65.2 | 117797773 |
| DCA2F5 | AC2 | AC | 42087722 | 27417036 | 65.1 | 104456992 |
| DAC2F6 | AC2 | AC | 52273616 | 33887203 | 64.8 | 129992828 |
| DAC3F1 | AC3 | AC | 36797308 | 23489713 | 63.8 | 92620173 |
| DAC3F2 | AC3 | AC | 37738469 | 24185609 | 64.1 | 94605573 |
| DAC3M1 | AC3 | AC | 42068037 | 27090733 | 64.4 | 98714774 |
| DAC3M2 | AC3 | AC | 39339493 | 25768196 | 65.5 | 95317572 |
| DAC4F1 | AC4 | AC | 42299679 | 27295026 | 64.5 | 104846314 |
| DAC4F2 | AC4 | AC | 38833941 | 25616722 | 66 | 91156715 |
| DAC4F3 | AC4 | AC | 44646462 | 28885046 | 64.7 | 108022783 |
| DAC4F4 | AC4 | AC | 35895025 | 23051704 | 64.2 | 86950894 |
| DAC4F5 | AC4 | AC | 39404821 | 25781843 | 65.4 | 97419679 |
| DAC5F1 | AC5 | AC | 35520790 | 22996317 | 64.7 | 91318066 |
| DAC5F2 | AC5 | AC | 34399347 | 22060676 | 64.1 | 86540817 |
| DAC5F4 | AC5 | AC | 43250989 | 27446249 | 63.5 | 111158104 |
| DAC5F5 | AC5 | AC | 32067081 | 20329992 | 63.4 | 79084872 |
| DAC5M1 | AC5 | AC | 51334217 | 33079315 | 64.4 | 128898302 |
| DAC5M2 | AC5 | AC | 33883102 | 21546393 | 63.6 | 87035290 |
| DAC5M3 | AC5 | AC | 52958732 | 33252171 | 62.8 | 130395005 |
| DAC5M4 | AC5 | AC | 44501037 | 28021901 | 63 | 112560791 |
| DAC6F1 | AC6 | AC | 33528816 | 21593698 | 64.4 | 84081178 |
| DAC6F2 | AC6 | AC | 50372373 | 31789055 | 63.1 | 129605132 |
| DAC6F3 | AC6 | AC | 32607409 | 21187621 | 65 | 81787453 |
| DAC6M1 | AC6 | AC | 41774175 | 27114116 | 64.9 | 106145628 |
| DAC6M2 | AC6 | AC | 41709069 | 27163395 | 65.1 | 105006347 |
| DAC6M3 | AC6 | AC | 37751102 | 24179403 | 64 | 96247756 |
| DAC7F1 | AC7 | AC | 40932521 | 26085567 | 63.7 | 102525563 |
| DAC7F2 | AC7 | AC | 35159822 | 22676196 | 64.5 | 89150745 |
| DAC7F3 | AC7 | AC | 46822287 | 30262680 | 64.6 | 118016386 |
| DAC7F4 | AC7 | AC | 46845900 | 30222377 | 64.5 | 115263867 |
| DAC7F5 | AC7 | AC | 33502745 | 21346461 | 63.7 | 84679267 |
| DAC7F6 | AC7 | AC | 43014769 | 27896668 | 64.9 | 106490438 |
| DAC7M1 | AC7 | AC | 38875953 | 24934336 | 64.1 | 97924777 |
| DAC7M2 | AC7 | AC | 44036322 | 28590330 | 64.9 | 112034306 |
| DAC7M3 | AC7 | AC | 38351446 | 24245545 | 63.2 | 99605550 |
| DC2F1 | C2 | C | 40080369 | 26117911 | 65.2 | 97891545 |
| DC2F2 | C2 | C | 51182608 | 33490312 | 65.4 | 126498577 |
| DC2M1 | C2 | C | 45877796 | 29680391 | 64.7 | 114295937 |
| DC2M2 | C2 | C | 35245691 | 23025202 | 65.3 | 86418240 |
| DC3F1 | C3 | C | 46267701 | 30176938 | 65.2 | 113007343 |
| DC3F2 | C3 | C | 34932280 | 22514788 | 64.5 | 87265988 |
| DC3F3 | C3 | C | 33798394 | 22297077 | 66 | 85674852 |
| DC3M1 | C3 | C | 43007290 | 27926314 | 64.9 | 107756857 |
| DC3M2 | C3 | C | 48221046 | 31475567 | 65.3 | 116367445 |
| DC3M4 | C3 | C | 34537031 | 22427627 | 64.9 | 86087659 |
| DC4F1 | C4 | C | 41872520 | 26890972 | 64.2 | 107634598 |
| DC4F2 | C4 | C | 30430798 | 19407053 | 63.8 | 77707002 |
| DC4F3 | C4 | C | 27217203 | 16749896 | 61.5 | 68905729 |
| DC4F4 | C4 | C | 46664303 | 29303278 | 62.8 | 118628026 |
| DC4F5 | C4 | C | 48528470 | 30773105 | 63.4 | 122099069 |
| DC4M1 | C4 | C | 45503310 | 29476316 | 64.8 | 111721612 |
| DC4M2 | C4 | C | 37935125 | 24288489 | 64 | 95517747 |
| DC5F1 | C5 | C | 41053179 | 26957837 | 65.7 | 95666236 |
| DC5F2 | C5 | C | 52350155 | 34444682 | 65.8 | 122965840 |
| DC5F3 | C5 | C | 48895800 | 31818037 | 65.1 | 124246339 |
| DC5F4 | C5 | C | 49888065 | 32290486 | 64.7 | 127533130 |
| DC5F5 | C5 | C | 47124142 | 30051078 | 63.8 | 118180337 |
| DC5M1 | C5 | C | 32382454 | 20366410 | 62.9 | 82841447 |
| DC5M2 | C5 | C | 45268986 | 29236019 | 64.6 | 114060739 |
| DC5M3 | C5 | C | 39444571 | 25275174 | 64.1 | 100206546 |
| DC6F1 | C6 | C | 40219715 | 25115022 | 62.4 | 101955535 |
| DC6F2 | C6 | C | 42812741 | 27643746 | 64.6 | 107588058 |
| DC6F3 | C6 | C | 38439005 | 24284023 | 63.2 | 100658041 |
| DC6F4 | C6 | C | 37942458 | 24238843 | 63.9 | 97366540 |
| DC6M1 | C6 | C | 48344192 | 30463474 | 63 | 123522809 |
| DC6M2 | C6 | C | 39951057 | 25828554 | 64.7 | 101770343 |
| DC6M4 | C6 | C | 45500790 | 29625204 | 65.1 | 116278147 |
| DC7F1 | C7 | C | 38888852 | 24886492 | 64 | 100326536 |
| DC7F2 | C7 | C | 36797134 | 23780841 | 64.6 | 93471728 |
| DC7F3 | C7 | C | 35959914 | 23117711 | 64.3 | 91641930 |
| DC7M1 | C7 | C | 32744548 | 20802911 | 63.5 | 84946407 |
| DC7M2 | C7 | C | 34459262 | 21591368 | 62.7 | 86818179 |
| DC7M3 | C7 | C | 37871806 | 24327435 | 64.2 | 98180509 |
| DC7M4 | C7 | C | 35014113 | 22055170 | 63 | 89874117 |
| DC7M5 | C7 | C | 38317384 | 24378675 | 63.6 | 98536600 |

**Supplemental Table 3. Results of linear mixed models of effect of alarm cue versus control cue on behavioural measurements.**

|  | Estimate | Std. Error | R^2^ |
| --- | --- | --- | --- |
| *Activity (Distance travelled)* |  |  | 0.307 |
| Intercept | 452.05 | 122.34 |  |
| Cue:control | 76.64 | 72.84 | 0.021 |
| Sex:male | -14.46 | 188.99 | 0.000 |
| Mass | 336.93 | 817.58 | 0.002 |
| Sex:male * Mass | 4323.99 | 2012.23 | 0.055 |
|  |  |  |  |
| *Boldness (Time in shelter or frozen)* |  |  | 0.040 |
| Intercept | 192.81 | 29.33 |  |
| Cue:control | 13.24 | 15.27 | 0.09 |
| Sex:male | 28.59 | 19.26 | 0.027 |
| Mass | 144.82 | 196.28 | 0.007 |
|  |  |  |  |
| *Exploration (Squares explored)* |  |  | 0.067 |
| Intercept | 2.85 | 0.11 |  |
| Cue:control | -0.06 | 0.07 | 0.014 |
| Sex:male | 0.16 | 0.07 | 0.055 |
| Mass | 0.64 | 0.72 | 0.008 |
|  |  |  |  |
| *Shoaling (Preference for shoal)* |  |  | 0.148 |
| Intercept | 31.00 | 9.86 |  |
| Cue:control | -33.46 | 12.92 | 0.116 |
| Sex:male | 24.18 | 10.01 | 0.063 |

Tank was included as a random effect in all models. n = 81, 38 alarm cue fish and 41 control fish.

**Supplemental Table 4. Full linear mixed model results for associations between differentially methylated regions (DMSs) and shoaling.**

|  | Estimate | Std. Error | X^2^ | df | *p* | R^2^ |
| --- | --- | --- | --- | --- | --- | --- |
| Females - Shoaling |  |  |  |  |  | 0.608 |
| (Intercept) | 5.176 | 9.598 |  |  |  |  |
| NC_024335.1_32164113 | -9.827 | 5.971 | 2.708 | 1 | 0.100 | 0.056 |
| NC_024337.1_29659440 | 7.217 | 6.878 | 1.101 | 1 | 0.294 | 0.023 |
| NC_024340.1_31459107 | 10.017 | 6.871 | 2.125 | 1 | 0.145 | 0.048 |
| NC_024346.1_20253909 | -10.474 | 6.879 | 2.319 | 1 | 0.128 | 0.046 |
| NC_024346.1_24973192 | -5.719 | 7.933 | 0.520 | 1 | 0.471 | 0.011 |
| NC_024346.1_26750001 | 6.350 | 7.580 | 0.702 | 1 | 0.402 | 0.014 |
| **NC_024347.1_3570681** | -11.737 | 5.883 | 3.980 | 1 | **0.046** | 0.077 |
| NC_024350.1_13707429 | 5.731 | 6.178 | 0.861 | 1 | 0.354 | 0.019 |
| NC_024352.1_979014 | -5.492 | 5.674 | 0.937 | 1 | 0.333 | 0.020 |
| **NC_024352.1_6869839** | 15.766 | 5.434 | 8.419 | 1 | **0.004** | 0.154 |
| Cue:C | 15.361 | 12.445 | 1.777 | 1 | 0.183 | 0.000 |
|  |  |  |  |  |  |  |
| Males - Shoaling |  |  |  |  |  | 0.726 |
| (Intercept) | 33.509 | 11.453 |  |  |  |  |
| NC_024331.1_25505925 | 12.060 | 7.519 | 2.572 | 1 | 0.109 | 0.081 |
| NC_024333.1_34892207 | 2.845 | 10.036 | 0.080 | 1 | 0.777 | 0.003 |
| NC_024340.1_4699900 | 8.670 | 7.949 | 1.190 | 1 | 0.275 | 0.039 |
| NC_024341.1_18557385 | -12.612 | 6.806 | 3.433 | 1 | 0.064 | 0.106 |
| NC_024342.1_14034423 | -12.250 | 8.590 | 2.034 | 1 | 0.154 | 0.065 |
| **NC_024344.1_21712760** | 15.996 | 6.651 | 5.784 | 1 | **0.016** | 0.166 |
| NC_024345.1_9020419 | 2.271 | 6.949 | 0.107 | 1 | 0.744 | 0.004 |
| NC_024345.1_21381074 | 13.224 | 7.714 | 2.939 | 1 | 0.086 | 0.091 |
| NC_024353.1_7384371 | 7.084 | 7.738 | 0.838 | 1 | 0.360 | 0.028 |
| NC_024353.1_7579329 | -2.923 | 9.436 | 0.096 | 1 | 0.757 | 0.003 |
| Cue:C | 0.917 | 17.015 | 0.003 | 1 | 0.957 | 0.000 |

Significant *p*-values are bolded.

**Supplemental Table 5. G test results for the proportion of differentially methylated sites (DMSs) and regions (DMRs) distributed in genomic features compared to a null distribution.**

|  | Test | G | df | *p*-value | Direction of Change |
| --- | --- | --- | --- | --- | --- |
| Females - DMS | Initial | 346.26 | 3 | <0.0001 |  |
|  | Promoters | 7.08 | 1 | 0.008 | Decrease |
|  | Exons | 327.11 | 1 | <0.0001 | Decrease |
|  | Introns | 42.59 | 1 | <0.0001 | Increase |
|  | Intergenic | 35.95 | 1 | <0.0001 | Increase |
| Males - DMS |  |  |  |  |  |
|  | Initial | 46.69 | 3 | <0.0001 |  |
|  | Promoters | 7.69 | 1 | <0.0001 | Decrease |
|  | Exons | 902.39 | 1 | <0.0001 | Decrease |
|  | Introns | 29.72 | 1 | <0.0001 | Increase |
|  | Intergenic | 217.74 | 1 | <0.0001 | Increase |

Initial = initial G-test for difference in overall distributions. Subsequent G-tests done for each type of genomic feature. df = degrees of freedom

**Supplemental Figure 1. Schematics of behavioural assays, plan views.** The first behavioural assay was a modified open field test with a 10 cm x 10 cm artificial lawn aquarium plant that fish could hide in placed in one corner of the tank, represented by a green square. Additionally, a 4 x 8 virtual grid was overlayed onto the arena. The second assay was a shoaling test with two identical glass cylinders with a 9 cm diameter placed on each side of the tank: one empty, and the other containing a shoal of four unfamiliar adult females from the Paria population.

**Supplemental Figure 2. Heatmaps of differentially methylated sites (DMS) for (A) females and (B) males.** Heatmap of DMS with hierarchical clustering of samples for (A) females and (B) males. Each row is a DMS and each column is an individual. Scaled percent methylation for each DMS is displayed in heatmap.

**Supplemental Figure 3. Linear mixed models showing association between percent methylation at specific differentially methylated regions (DMRs) and shoaling in (A) females and (B to D) males.** Percent methylation was mean centered. Shoaling is measured as preference for the shoal in a shoaling test. DMRs were selected for analysis by elastic net regression. Linear mixed models were run separately for each sex with shoaling as the dependent variable and methylation at elastic net selected DMRs as the predictor variables. Tank was included as a random effect. Only DMRs *with* *p* < 0.1 are shown. *p* – values of DMRs in models shown on plot.

**Supplemental Figure 4. Manhattan plots of differentially methylated sites (DMS) for (A) females and (B) males.** Chromosome 12 is the sex chromosome.

**
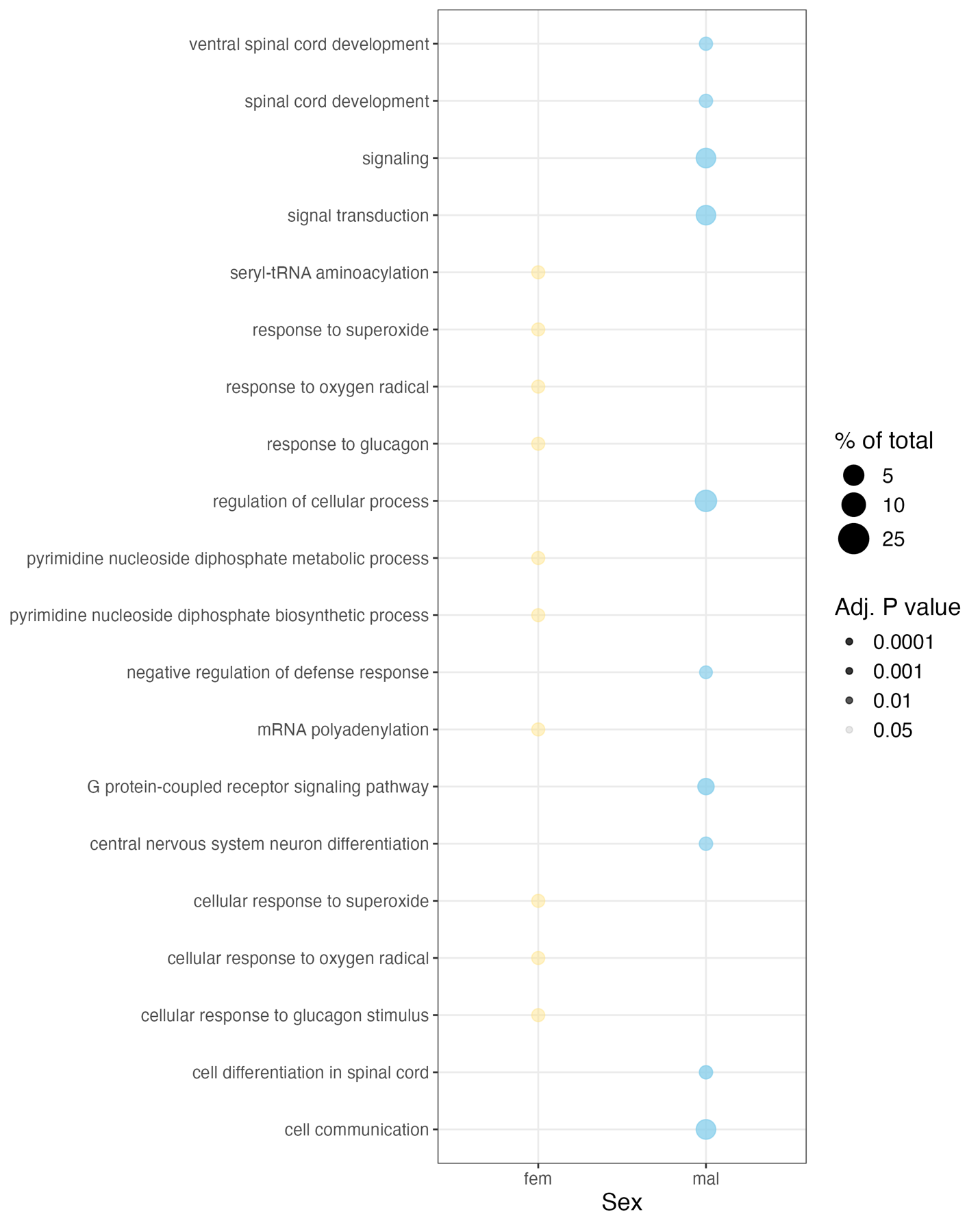
**

**Supplemental Figure 5. Gene ontology enrichment analysis results for hypermethylated differentially methylated sites (DMS) for females and males.**

**
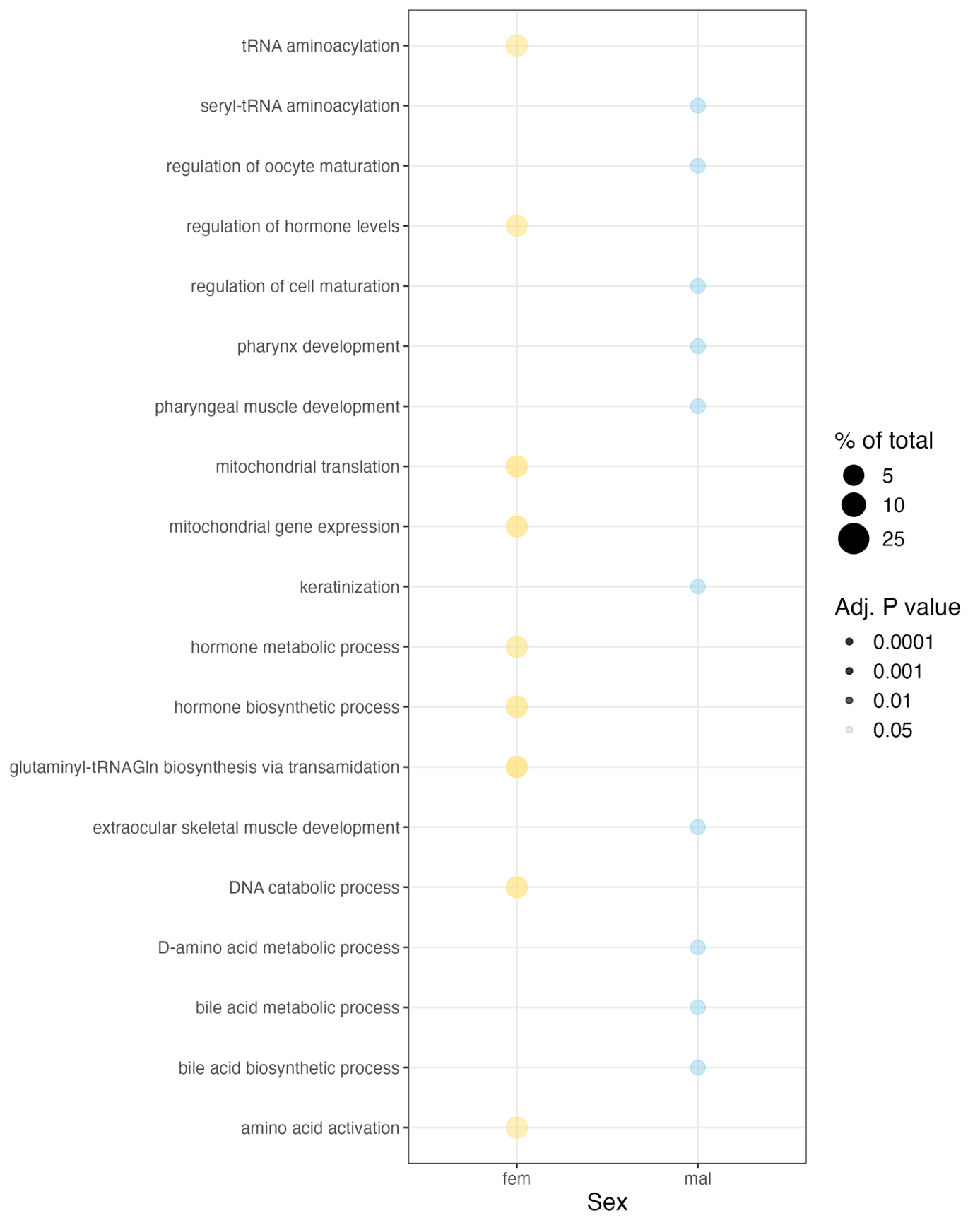
**

**Supplemental Figure 6. Gene ontology enrichment analysis results for hypermethylated differentially methylated regions (DMR) for females and males.**

**
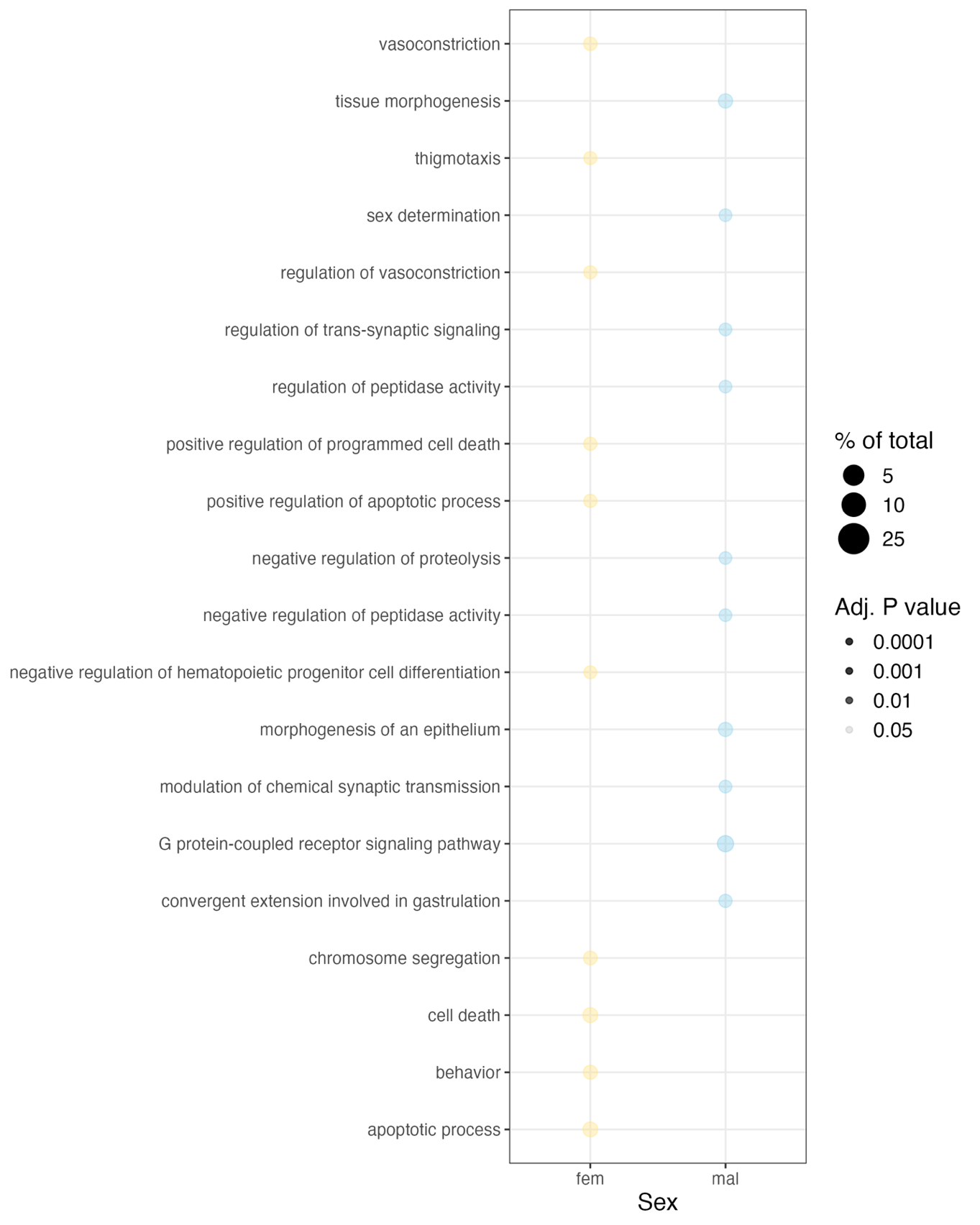
**

**Supplemental Figure 7. Gene ontology enrichment analysis results for hypomethylated differentially methylated regions (DMS) for females and males.**

**
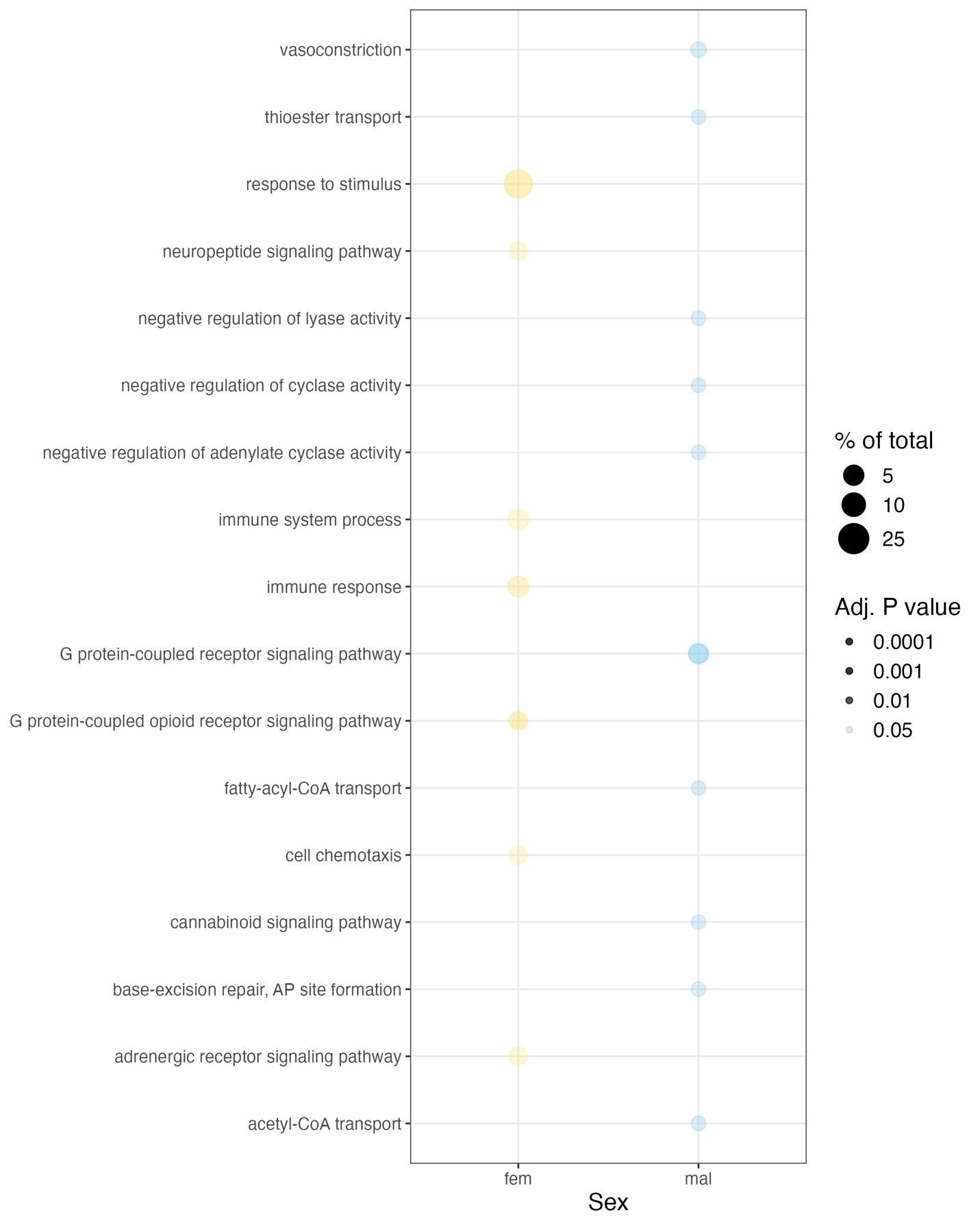
**

**Supplemental Figure 8. Gene ontology enrichment analysis results for hypomethylated differentially methylated regions (DMR) for females and males.**
